# Bimetallic iron–iron and iron–zinc complexes of the redox-active ONO pincer ligand†
†Electronic supplementary information (ESI) available: Complete experimental procedures and magnetic measurements and models. CCDC 1417565–1417567. For ESI and crystallographic data in CIF or other electronic format see DOI: 10.1039/c5sc03006d



**DOI:** 10.1039/c5sc03006d

**Published:** 2015-12-08

**Authors:** Janice L. Wong, Robert F. Higgins, Indrani Bhowmick, David Xi Cao, Géza Szigethy, Joseph W. Ziller, Matthew P. Shores, Alan F. Heyduk

**Affiliations:** a Department of Chemistry , University of California , Irvine , California 92697 , USA . Email: aheyduk@uci.edu ; Tel: +1-949-824-8806; b Department of Chemistry , Colorado State University , Fort Collins , Colorado 80523 , USA . Email: matthew.shores@colostate.edu ; Tel: +1-970-491-7235

## Abstract

A new bimetallic platform comprising a six-coordinate Fe(ONO)_2_ unit bound to an (ONO)M (M = Fe, Zn) has been discovered ((ONO^cat^)H_3_ = bis(3,5-di-*tert*-butyl-2-phenol)amine). Reaction of Fe(ONO)_2_ with either (ONO^cat^)Fe(py)_3_ or with (ONO^q^)FeCl_2_ under reducing conditions led to the formation of the bimetallic complex Fe_2_(ONO)_3_, which includes unique five- and six-coordinate iron centers. Similarly, the reaction of Fe(ONO)_2_ with the new synthon (ONO^sq^˙)Zn(py)_2_ led to the formation of the heterobimetallic complex FeZn(ONO)_3_, with a six-coordinate iron center and a five-coordinate zinc center. Both bimetallic complexes were characterized by single-crystal X-ray diffraction studies, solid-state magnetic measurements, and multiple spectroscopic techniques. The magnetic data for FeZn(ONO)_3_ are consistent with a ground state *S* = 3/2 spin system, generated from a high-spin iron(ii) center that is antiferromagnetically coupled to a single (ONO^sq^˙)^2–^ radical ligand. In the case of Fe_2_(ONO)_3_, the magnetic data revealed a ground state *S* = 7/2 spin system arising from the interactions of one high-spin iron(ii) center, one high-spin iron(iii) center, and two (ONO^sq^˙)^2–^ radical ligands.

## Abstract 2

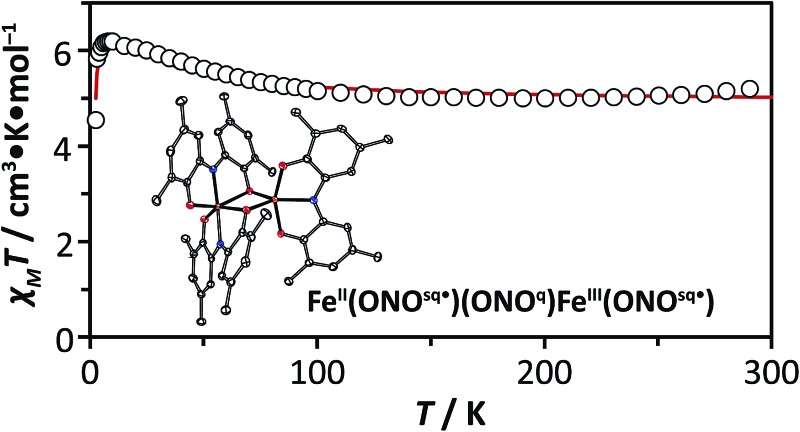
Synthetic and magnetic studies of homo- and heterobimetallic complexes of the redox-active ONO pincer ligand.

## Introduction

The controlled, modular assembly of multi-metallic coordination complexes with novel structures, electronic and magnetic properties, and reactivities remains a challenge in synthetic inorganic chemistry. Well-tailored multidentate ligands have been used to great effect in the synthesis of small homo- and heterometallic clusters.^1,2^ Ligands that feature differentiated metal binding sites have been used to access heterobimetallic complexes with two different metal ions and with unexpected reactivity profiles.^3,4^ Metallocene complexes have been elaborated into multi-metallic complexes through the incorporation of ancillary metal binding groups into the cyclopentadienyl ligand.^5,6^ These efforts typically aim to exploit the redox properties of the metallocene in the resulting multi-metallic assembly. Our strategy takes a modular approach in which a discrete metal complex of a redox-active pincer ligand is used as a ligand for a second metal center to generate homo- and heterobimetallic complexes.

**Chart 1:**
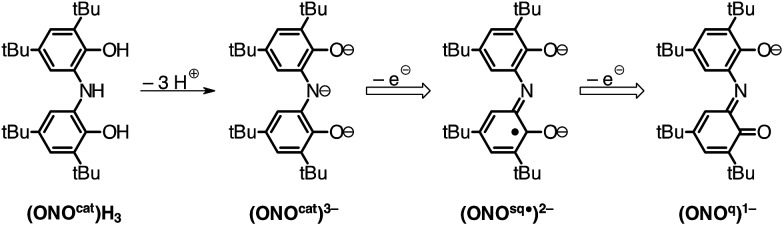
Oxidation states of the ONO pincer ligand.

The redox-active ONO pincer ligand, derived from bis(3,5-di-*tert*-butyl-2-phenol)amine ((ONO^cat^)H_3_; Chart 1), has received considerable attention for the preparation of coordination complexes with interesting electronic structures and unusual reactivities. A myriad of homoleptic, six-coordinate compounds of the formula M(ONO)_2_ have been reported in the literature for both 3d metal ions and a variety of main group metals.^7–12^ While many of these complexes display multiple stable oxidation states, their saturated coordination sphere limits their ability to perform inner-sphere reactivity.

We have discovered that the M(ONO)_2_ fragment itself can be used as a building block for the preparation of multimetallic complexes. The well-known complex Fe(ONO)_2_ can act as a chelating ligand towards a second metal center through the formation of two μ^2^-phenolate bridging units. New homobimetallic Fe_2_(ONO)_3_ and heterobimetallic FeZn(ONO)_3_ complexes have been prepared and their electronic and magnetic properties studied. The results reported here highlight both a modular method for preparing bimetallic structures and the electronic and magnetic complexity that arises from the combination of radical ligands and multiple open-shell metal ions.

## Results and discussion

### Synthesis and structural characterization

**Fig. 1:**
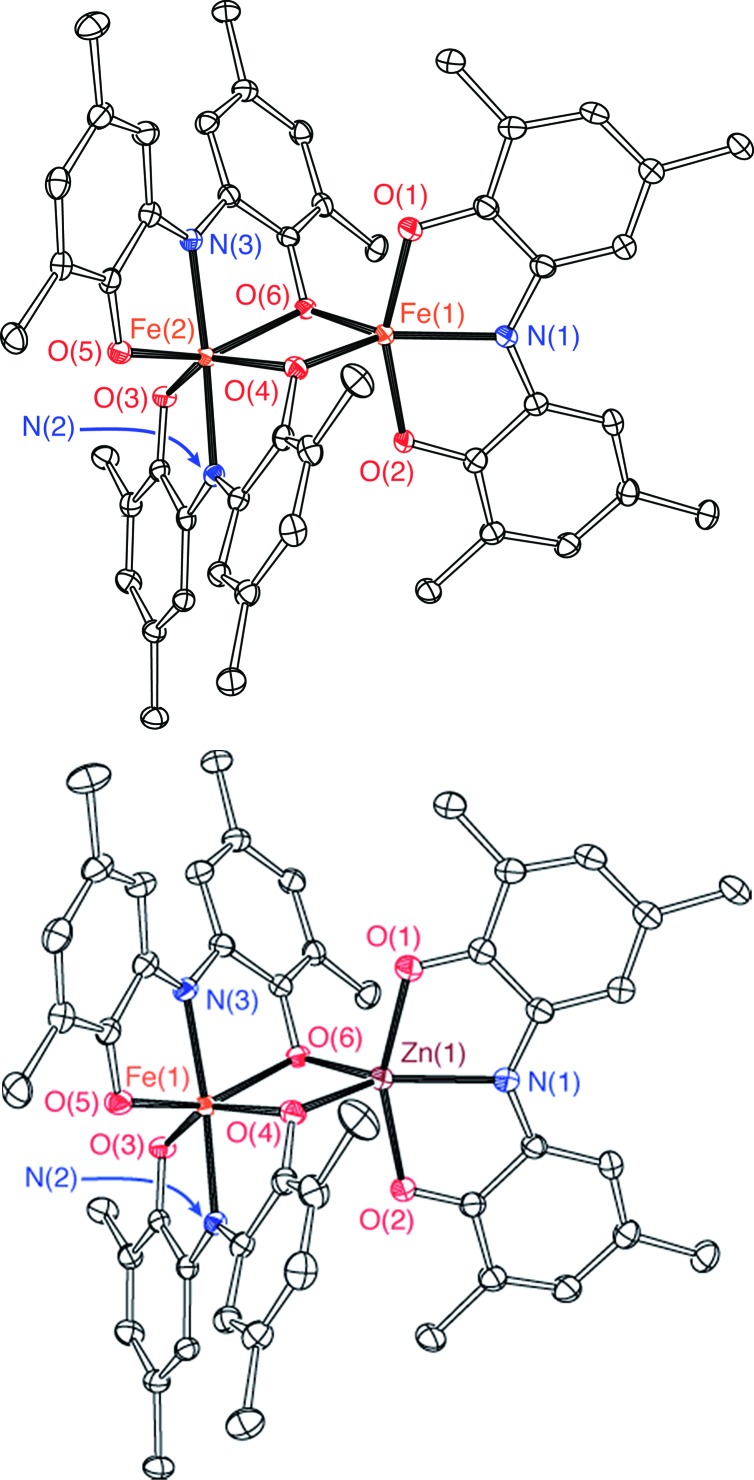
ORTEP diagrams of Fe_2_(ONO)_3_ (top) and FeZn(ONO)_3_ (bottom). Ellipsoids are drawn at 50% probability. Hydrogen atoms, methyl carbons of *tert*-butyl groups, and solvent molecules are omitted for clarity.

The bimetallic complex Fe_2_(ONO)_3_ was initially isolated in low yields as a crystalline solid upon treatment of (ONO^q^)FeCl_2_ (ref. 13) with KC_8_ in the absence of a strongly coordinating solvent. The bimetallic complex was identified and structurally characterized by single-crystal X-ray diffraction experiments. Purple crystals of Fe_2_(ONO)_3_, isolated by the Pasteur method, were monoclinic, falling into the space group *P*2_1_/*c*. The structure of Fe_2_(ONO)_3_ is given as an ORTEP diagram in Fig. 1, top. The complex comprises two different iron centers. One iron, Fe(1), is five-coordinate with *τ* = 0.265 (eqn (1); *α* = ∠N(1)–Fe(1)–O(6) = 140.64(6)°; *β* = ∠O(1)–Fe(1)–O(2) = 156.53(6)°), indicating a geometry that is intermediate between trigonal bipyramidal (*τ* = 1) and square pyramidal (*τ* = 0).^14^
1
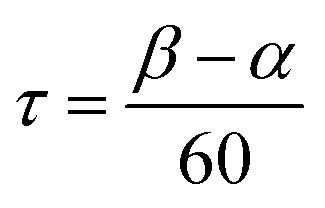
 Three of the coordination sites in the five-coordinate fragment are filled by a planar ONO ligand with two equatorial coordination sites occupied by oxygen donors from the neighboring six-coordinate iron fragment, Fe(2). This six-coordinate iron fragment adopts a pseudo-octahedral coordination environment owing to the meridional binding of two ONO ligands. The Fe(1)···Fe(2) separation is 3.07 Å, which falls well outside of the covalent radii of two iron atoms (2 × 116 pm), is too long for a formal metal–metal bond.^15^

Assignment of the metal oxidation state for the five-coordinate iron center can be made by comparing the structural metrics for Fe(1) of Fe_2_(ONO)_3_ with the previously published structures (ONO^q^)Fe^III^Cl_2_ and (ONO^cat^)Fe^III^(py)_3_.^13^ Whereas iron–oxygen bond distances appear to be insensitive to the oxidation state of the ONO ligand, the iron–nitrogen bond distance is shorter for reduced forms of the ONO ligand. Accordingly, (ONO^q^)Fe^III^Cl_2_ has an Fe–N distance of 2.1571(14) Å whereas (ONO^cat^)Fe^III^(py)_3_ has an Fe–N distance of 1.9902(18) Å. The iron–nitrogen bond distance, Fe(1)–N(1), of Fe_2_(ONO)_3_ falls between these two values at 2.0733(17) Å. Similarly, C–O and C–N bond distances within the non-bridging ONO ligand of the five-coordinate fragment are 1.316(2) and 1.374(3) Å, respectively, which fall in between the values measured for the C–O and C–N bond distances of (ONO^q^)Fe^III^Cl_2_ (shorter bonds) and (ONO^cat^)Fe^III^(py)_3_ (longer bonds). These results are consistent with the (ONO^sq^˙)^2–^ oxidation state for the non-bridging ONO ligand coordinated to Fe(1). Iron–oxygen bond distances to the bridging phenoxide donors are significantly shorter for the five-coordinate iron (average Fe(1)–(μ-O) = 1.976 ± 0.002 Å) than for the six-coordinate iron (average Fe(2)–(μ-O) = 2.162 ± 0.003 Å). Altogether, these data suggest that the five-coordinate iron center is best described as an [(ONO^sq^˙)Fe^III^]^1+^ fragment, with a six-coordinate [Fe(ONO)_2_]^1–^ fragment acting as a monoanionic chelating ligand to the Fe(1) center.

Definitive assignment of the metal oxidation state for the six-coordinate iron center, Fe(2), is not possible from the structural data alone. The neutral Fe(ONO)_2_ monometallic unit has been characterized as an iron(iii) complex containing one (ONO^sq^˙)^2–^ ligand and one (ONO^q^)^1–^ ligand.^16^ Addition of one electron to neutral Fe(ONO)_2_ to give the monoanion posited above (*i.e.* [Fe(ONO)_2_]^1–^), would then afford one of two limiting electron configurations:^16^ (a) assigning the added electron to the iron center would give [Fe^II^(ONO^sq^˙)(ONO^q^)]^1–^ or (b) assigning the added electron to the redox-active ligands would give [Fe^III^(ONO^sq^˙)_2_]^1–^. The structural data for Fe_2_(ONO)_3_ alone do not distinguish between these two possibilities. While intraligand bond distances for the two ONO ligands are the same within error, this result could stem from the symmetric [Fe^III^(ONO^sq^˙)_2_]^1–^ electronic configuration or from a mixed-valence [Fe^II^(ONO^sq^˙)(ONO^q^)]^1–^ electronic configuration with strong delocalization (class III Robin–Day mixed valency).^17,18^ More insight into the electronic configuration around the six-coordinate iron center was provided by analysis of the solid-state magnetic susceptibility data and solution electronic absorption data (*vide infra*).

**Scheme 1:**
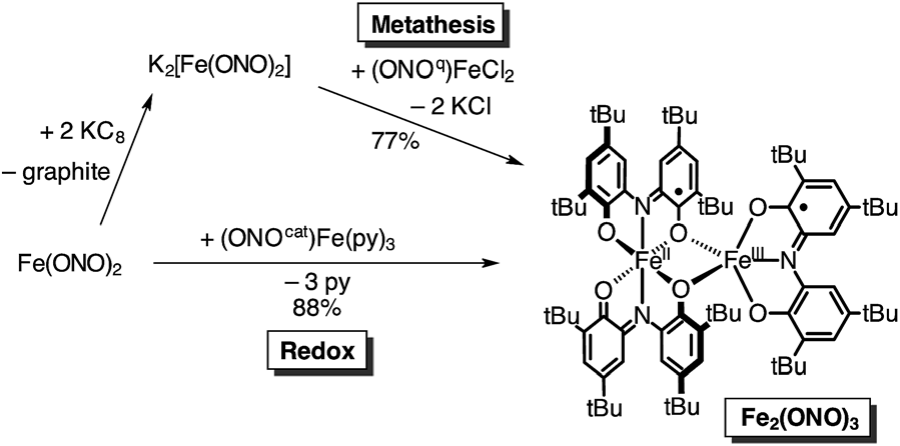
Synthesis of Fe_2_(ONO)_3_ by metathesis and redox pathways.

Given the novel structure and electronic properties of Fe_2_(ONO)_3_, a more direct synthetic method for accessing the complex was sought. Initial efforts were directed towards a metathesis route to prepare Fe_2_(ONO)_3_ from doubly-reduced Fe(ONO)_2_ and (ONO^q^)Fe^III^Cl_2_. According to Scheme 1, Fe(ONO)_2_ dissolved in THF was treated with two equivalents of freshly-prepared KC_8_, followed by one equivalent of (ONO^q^)Fe^III^Cl_2_. The product was isolated by extraction into toluene after removal of the THF solvent. Diffusion of acetonitrile into a toluene solution of the complex afforded the product as a dark purple microcrystalline solid in 77% yield. An alternative route to prepare Fe_2_(ONO)_3_ relies on the reaction between neutral Fe(ONO)_2_ and (ONO^cat^)Fe^III^(py)_3_. As summarized in Scheme 1, one-to-one mixtures of Fe(ONO)_2_ and (ONO^cat^)Fe^III^(py)_3_ in toluene produced the desired bimetallic complex in 88% yield. In this reaction, (ONO^cat^)Fe^III^(py)_3_ becomes the five-coordinate iron center after the expulsion of the pyridine ligands and the transfer of one electron to give the six-coordinate [Fe(ONO)_2_]^1–^ fragment. The order of the pyridine exchange and electron transfer reactions is not known; however, previous studies indicated that pyridine dissociation from (ONO^cat^)Fe^III^(py)_3_ occurs readily in solution.^13^

**Scheme 2:**
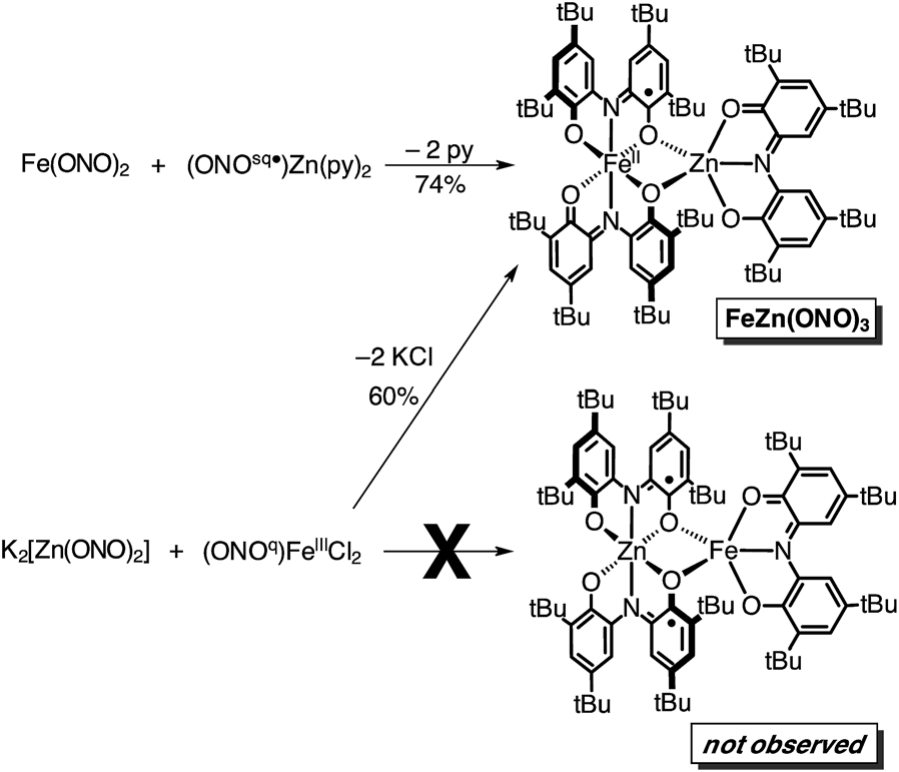
Synthesis of FeZn(ONO)_3_.

In an attempt to elaborate the bimetallic motif of Fe_2_(ONO)_3_, and in an attempt to generate a derivative with a simpler electronic structure, we targeted analogous zinc complexes. Efforts to prepare the all-zinc complex, *i.e.*, Zn_2_(ONO)_3_, were not successful. Alternatively, the new heterobimetallic complex FeZn(ONO)_3_, which contains a six-coordinate iron and a five-coordinate zinc, was prepared in high yields by the reaction of (ONO^sq^˙)Zn(py)_2_ with Fe(ONO)_2_ in toluene as summarized in Scheme 2. The new zinc synthon, (ONO^sq^˙)Zn(py)_2_, was prepared in 71% yield from zinc acetate and one equivalent of K_2_(ONO^sq^˙) in neat pyridine (eqn (2)). Attempts to prepare the complementary heterobimetallic complex containing a six-coordinate zinc and a five-coordinate iron, either from Zn(ONO)_2_ and (ONO^cat^)Fe^III^(py)_3_ or from [Zn(ONO)_2_]^2–^ and (ONO^q^)Fe^III^Cl_2_, produced only the isomer with a six-coordinate iron and five-coordinate zinc.2
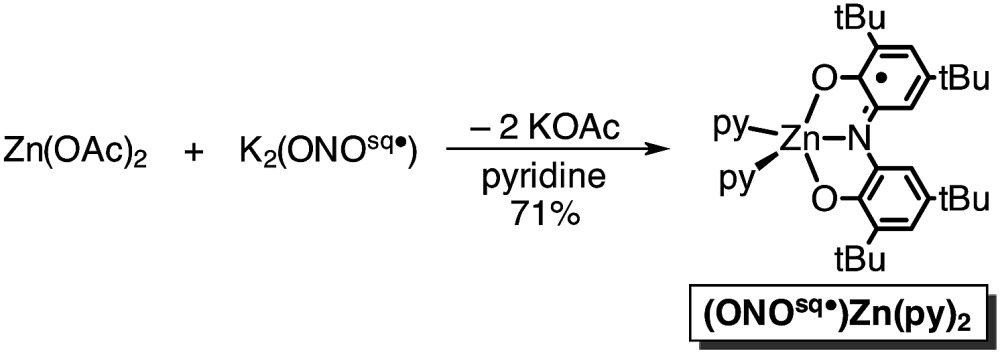



**Fig. 2:**
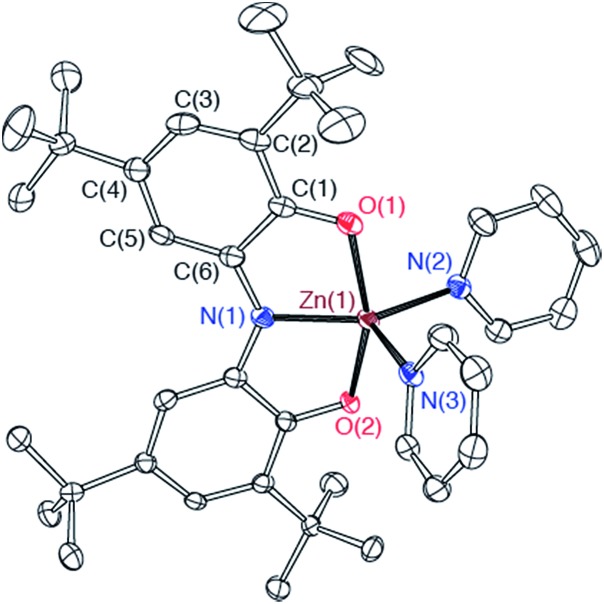
ORTEP diagram of one crystallographically unique (ONO^sq^˙)Zn^II^(py)_2_ molecule. Ellipsoids are drawn at 50% probability. Hydrogen atoms and pyridine and acetonitrile solvent molecules are omitted for clarity.

Both the monometallic (ONO^sq^˙)Zn^II^(py)_2_ complex and the heterobimetallic FeZn(ONO)_3_ complex were characterized by single-crystal X-ray diffraction studies. The structure of (ONO^sq^˙)Zn^II^(py)_2_ is presented as an ORTEP diagram in Fig. 2. The complex crystallizes in the *P*2_1_ space group and the asymmetric unit contains two (ONO^sq^˙)Zn^II^(py)_2_ molecules, one pyridine molecule, and one acetonitrile molecule. Unlike the square-planar geometry determined for (ONO^sq^˙)Zn^II^(NEt_3_),^19^ the structure of (ONO^sq^˙)Zn^II^(py)_2_ is five-coordinate with a geometry that lies in between square pyramidal and trigonal bipyramidal.^20^ Intraligand C–O and C–N bond distances of 1.307(5) Å and 1.358(5) Å, respectively, are consistent with the (ONO^sq^˙)^2–^ oxidation state of the redox-active ligand. The zinc–nitrogen bond distances to the pyridine ligands all fall in the 2.06–2.07 Å range. Consistent with a zinc(ii) coordinated to an (ONO^sq^˙)^2–^ ligand radical, the solution EPR spectrum of (ONO^sq^˙)Zn^II^(py)_2_ at 77 K shows a broad, isotropic signal at *g* = 2.00, consistent with an *S* = 1/2 spin system (see ESI†).

Single-crystal X-ray diffraction experiments performed on FeZn(ONO)_3_ confirm a solid-state structure analogous to Fe_2_(ONO)_3_ but with zinc occupying the five-coordinate metal site. Fig. 1, bottom, shows the structure of FeZn(ONO)_3_ as an ORTEP diagram. The Fe···Zn distance of 3.05 Å is too long for a metal–metal bond (the sum of their covalent radii is 234 pm). The five coordinate zinc center again lies between trigonal bipyramidal and square pyramidal geometries with *τ* = 0.252.^21^ The (ONO) ligand of the five-coordinate zinc center has contracted C–O bond distances of 1.266(3) Å and C–N bond distances of 1.342(3) Å, indicative of a monoanionic (ONO^q^)^1–^ ligand. Congruously, the C–C bond distances of this ONO ligand show strong cyclohexadiene character in the ligand backbone. For charge-balance considerations, the six-coordinate iron fragment must again act as a monoanionic chelating ligand towards the zinc center, with the same two possible oxidation states, either (a) [Fe^II^(ONO^sq^˙)(ONO^q^)]^1–^ or (b) [Fe^III^(ONO^sq^˙)_2_]^1–^. Again in this case, the structural data does not distinguish between these two possibilities; however, bond distances within the [Fe(ONO)_2_]^1–^ fragments of Fe_2_(ONO)_3_ and FeZn(ONO)_3_ show a high degree of similarity.

To provide further evidence for the identities of the metal ions in the five- and six-coordinate sites of FeZn(ONO)_3_, the complex was decomposed in neat pyridine as summarized in eqn (3). The ^1^H NMR spectrum of samples of FeZn(ONO)_3_ heated in pyridine-*d*_5_ revealed the presence of Fe(ONO)_2_ while no Zn(ONO)_2_ was observed. Similarly, an EPR spectrum of the reaction mixture displayed the isotropic, *S* = 1/2 signal indicating the formation of (ONO^sq^˙)Zn^II^(py)_2_, whereas no resonances associated with (ONO^cat^)Fe^III^(py)_3_ were observed. These results are consistent with iron occupying the six-coordinate site and zinc in the five-coordinate site as proposed by the single-crystal data. For comparison, heating samples of Fe_2_(ONO)_3_ in pyridine-*d*_5_ resulted in the generation of paramagnetic ^1^H NMR resonances for Fe(ONO)_2_ and EPR resonances for (ONO^cat^)Fe^III^(py)_3_.3
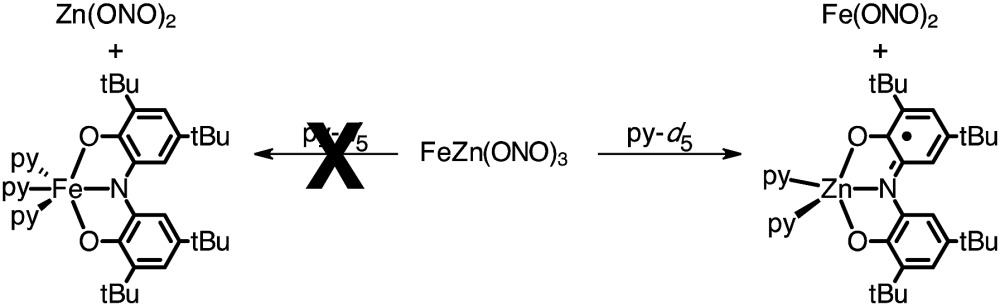



### Magnetism and electronic structure

In an effort to better elucidate the distribution of redox and spin states in Fe_2_(ONO)_3_ and FeZn(ONO)_3_, solid-state magnetic susceptibility experiments were carried out. Given the closed-shell electron configuration of zinc(ii) in FeZn(ONO)_3_, the magnetic behavior of the heterobimetallic complex was considered first. Fig. S7† shows *χ*_M_ *T* values for FeZn(ONO)_3_ between 2 K and 300 K. As the temperature decreases from 300 K, *χ*_M_ *T* decreases linearly from a value of 2.52 cm^3^ K mol^–1^ to a value of 1.59 cm^3^ K mol^–1^ at 10 K, consistent with temperature independent paramagnetism (TIP). Below 10 K, *χ*_M_ *T* drops abruptly to a value of 1.19 cm^3^ K mol^–1^ at 2 K, suggestive of magnetic anisotropy and/or antiferromagnetic coupling.

**Fig. 3:**
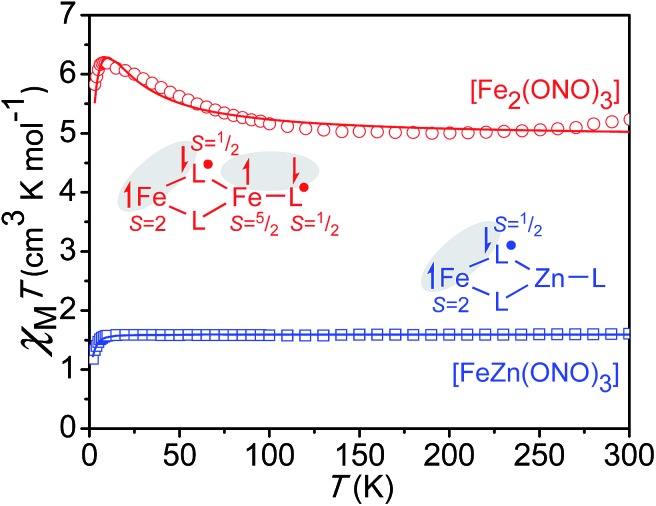
Temperature dependence of the dc magnetic susceptibility data for FeZn(ONO)_3_ (blue squares) and Fe_2_(ONO)_3_ (red circles) collected at an applied field of 1000 Oe; data have been corrected for TIP; the lines represent best fits to the models depicted in the inset schemes (julX).^22^

Given that the zinc and non-bridging ONO ligand can be treated as a closed-shell [Zn^II^(ONO^q^)]^1+^ fragment, two spin models were considered for modeling the magnetic susceptibility of FeZn(ONO)_3_. The first model (a) contained a high-spin Fe^II^ ion, a single (ONO^sq^˙)^2–^ ligand, and a single (ONO^q^)^1–^ ligand. The second model (b) contained a high-spin Fe^III^ ion and two (ONO^sq^˙)^2–^ ligands. In both scenarios, strong antiferromagnetic coupling between unpaired electrons on the iron and unpaired electrons on the ONO ligand(s) would generate an *S* = 3/2 total spin ground state and a theoretical *χ*_M_ *T* value of 1.875 cm^3^ K mol^–1^ with *g* = 2, consistent with the experimental value. The *S* = 3/2 system was modeled using julX^22^ to give the fit shown in Fig. 3 based on the following parameters: *g* = 1.84, axial anisotropy with *D* = –2.96 cm^–1^, TIP = 2.91 × 10^–3^ cm^3^ mol^–1^ (subtracted), and intermolecular coupling with *Θ* = –0.058 cm^–1^. A *g* value less than 2.0 is consistent with previous reports on the magnetic properties of Fe(ONO)_2_ ^8,16^ and consistent with the 4 K EPR spectrum of FeZn(ONO)_3_ shown in Fig. S3.† The TIP value is large but reasonable considering the accessibility of other electronic isomers and the proximity of spin excited states with larger *S* values.^23,24^ Further supporting this model, the magnetization trends toward saturation between 2–3 *μ*_B_ as the field is increased to 5 T at 1.8 K (Fig. S8†), consistent with *S* = 3/2 where *g* < 2 and significant axial anisotropy is observed. The PHI program^25^ was used to estimate the extent of antiferromagnetic coupling between the unpaired electrons on the iron and (ONO^sq^˙)^2–^ ligand(s) for the two spin models. As shown in Fig. S11,† the most reasonable parameters were found for model (a) that includes a high-spin Fe^II^ ion and only one (ONO^sq^˙)^2–^ ligand (*J* ≅ –260 cm^–1^). Notably model (b), which includes a high-spin Fe^III^ and two (ONO^sq^˙)^2–^ ligands, did not give realistic exchange coupling parameters (see ESI†).

Whereas the magnetic susceptibility data alone cannot definitively differentiate between the “high-spin Fe^II^ + one (ONO^sq^˙)^2–^” and “high-spin Fe^III^ + two (ONO^sq^˙)^2–^” electronic isomers, comparison of the electronic absorption spectra for FeZn(ONO)_3_ and Fe(ONO)_2_ lends support for the former arrangement. Fig. S5† shows the UV-vis-NIR absorption spectra of FeZn(ONO)_3_ and Fe(ONO)_2_. Both spectra show similar molar absorptivities for a band near 400 nm, which has been attributed to a semiquinonate-based transition.^26^ Since Fe(ONO)_2_ has been characterized as having one (ONO^sq^˙)^2–^ ligand, it follows that FeZn(ONO)_3_ has only one (ONO^sq^˙)^2–^ ligand. From the combined structural, magnetic, and electronic absorption data, we conclude that the FeZn(ONO)_3_ complex contains a high-spin ferrous ion and one bridging semiquinonate ligand. Thus, the ground state electron distribution in FeZn(ONO)_3_ can be assigned as Fe^II^(ONO^sq^˙)(ONO^q^)Zn^II^(ONO^q^), which is represented as a Lewis structure in Scheme 2.

**Fig. 4:**
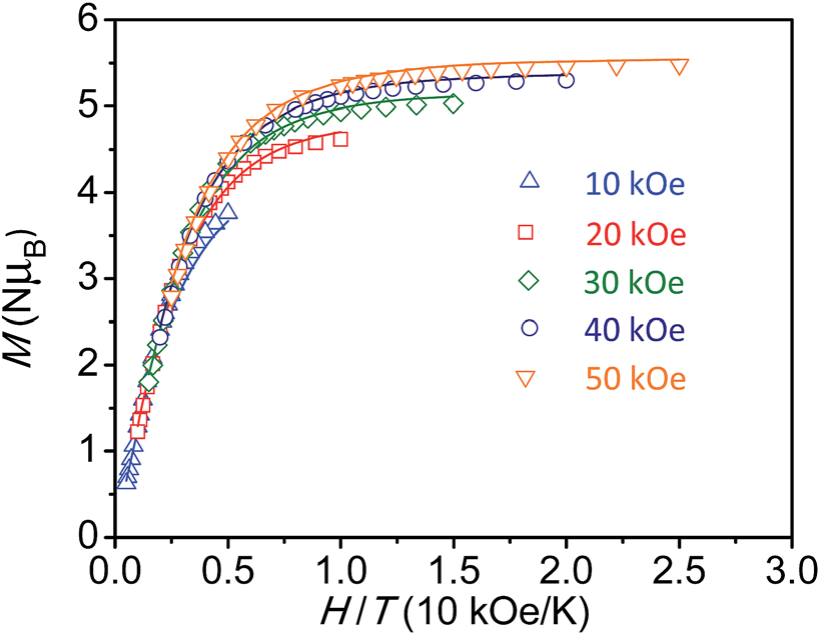
Reduced field dependence of magnetization for Fe_2_(ONO)_3_; lines represent best fits of the data to an *S* = 7/2 ground state model, as determined by ANISOFIT.^27^

For the diiron complex Fe_2_(ONO)_3_, the replacement of the diamagnetic (ONO^q^)Zn^II^ fragment with a paramagnetic (ONO^sq^˙)Fe^III^ fragment significantly changes the magnetic properties of the complex. The magnetization dependence on reduced field for Fe_2_(ONO)_3_, shown in Fig. 4, shows non-superimposable isofield lines and saturation of the magnetization at 5.48 *μ*_B_ in a 5 T magnetic field. Together, these data support an *S* = 7/2 ground state with significant axial magnetic anisotropy and *g* < 2. Attempts to fit the magnetization *vs.* applied magnetic field data to various spin state and anisotropy models using ANISOFIT^27^ are summarized in the ESI:† the best fit has the parameters *S* = 7/2, *g* = 1.95, *D* = 2.40 cm^–1^ and |*E*| = 0.00177 cm^–1^. Again, the low *g* value is consistent with the EPR spectrum of Fe_2_(ONO)_3_ collected at 4 K (Fig. S4†), which shows signals at *g* = 2.02, 1.96 and 1.86.

The magnetic susceptibility data for Fe_2_(ONO)_3_ support an electronic configuration that is best described as Fe^II^(ONO^sq^˙)(ONO^q^)Fe^III^(ONO^sq^˙). This configuration is represented as a Lewis structure in Scheme 1, and it preserves the same [Fe^II^(ONO^sq^˙)(ONO^q^)]^–^ six-coordinate unit that was postulated above for FeZn(ONO)_3_. Consistent with two different iron centers, the Mössbauer spectrum of Fe_2_(ONO)_3_ (Fig. S6†) shows two overlapping doublet signals.^28^ Fig. S12† shows the temperature-dependent magnetic susceptibility data for Fe_2_(ONO)_3_ at an applied field of 1000 Oe. At ambient temperature, *χ*_M_ *T* has a value of 7.00 cm^3^ K mol^–1^, which decreases to 5.76 cm^3^ K mol^–1^ at 100 K, then begins an upturn, achieving a maximum value of 6.25 cm^3^ K mol^–1^ at 10 K. Below 10 K, *χ*_M_ *T* decreases rapidly to 5.69 cm^3^ K mol^–1^ at 2.5 K. Attempts to model this data using unconstrained interactions among four spin centers (two iron centers and two (ONO^sq^˙)^2–^ ligands) failed to fit the data in a reasonable way. Instead, a simpler model was developed that explicitly conserves the six-coordinate, *S* = 3/2, Fe^II^(ONO^sq^˙)(ONO^q^) fragment from the FeZn(ONO)_3_ complex and combines it with a five-coordinate, *S* = 2, (ONO^sq^˙)Fe^III^ fragment suggested by the single-crystal structural data. The resulting fit to the *χ*_M_ *T* data for Fe_2_(ONO)_3_ is shown in Fig. 3 and provided the following parameters: *g*_3/2_ = 1.84, *g*_2_ = 2.10, *D* = 5.15 cm^–1^, |*E*| = 0.04 cm^–1^, *J* = +1.57 cm^–1^, and TIP = 2.59 × 10^–3^ cm^3^ mol^–1^ (subtracted). Further support for this model can be found in the EPR and electronic absorption spectra. In the UV-vis spectrum, we note that the diiron complex exhibits molar absorptivity values nearly twice as large as FeZn(ONO)_3_ near 400 nm (Fig. S5†), consistent with the presence of two ligand radicals that are not strongly coupled to one another. Other models considered (see ESI†) did not fit with the combined structural, spectroscopic and magnetic data.

## Conclusions

Formal reduction of Fe(ONO)_2_ affords a “ligand” with an electronic structure that is best described as [Fe^II^(ONO^sq^˙)(ONO^q^)]^–^, which can act as a monoanionic chelating ligand, allowing the construction of bimetallic complexes with interesting redox and magnetic properties. The [Fe^II^(ONO^sq^˙)(ONO^q^)]^–^ unit coordinates to a second metal center through the formation of two μ^2^-phenoxide oxygen atoms. For the iron–zinc complex, FeZn(ONO)_3_, the complex is best described as Fe^II^(ONO^sq^˙)(ONO^q^)Zn^II^(ONO^q^) with an *S* = 3/2 ground state. In this bimetallic complex, the juxtaposition of (ONO^q^)^–^ and (ONO^sq^˙)^2–^ ligands on the six-coordinate iron center sets up an example of ligand-based mixed-valency. In the case of the diiron complex, Fe_2_(ONO)_3_, the complex is best described as Fe^II^(ONO^sq^˙)(ONO^q^)Fe^III^(ONO^sq^˙) with an *S* = 7/2 ground state. The diiron complex is unusual in that it is an example of a double mixed-valence complex: in addition to the same ligand-based mixed valency, the juxtaposition of iron(ii) and iron(iii) centers with different coordination geometries affords metal-based mixed-valence character.^17^ The modular nature of these molecules suggests a new strategy for the synthesis of multi-metallic systems with intriguing redox and magnetic properties.
