# Supplementary material for: Bimetallic iron–iron and iron–zinc complexes of the redox-active ONO pincer ligand
Source: Chem Sci. 2015 Dec 8;7(2):1594–9. doi: 10.1039/c5sc03006d (PMC5530940; doi:10.1039/c5sc03006d)
Supplement: Supplementary file 1 [file SC-007-C5SC03006D-s001.pdf]

Electronic Supplementary Material (ESI) for Chemical Science.
This journal is © The Royal Society of Chemistry 2015

Bimetallic iron-iron and iron-zinc complexes of the redox-active ONO pincer ligand

Janice L. Wong,[a] Robert F. Higgins,[b] Indrani Bhowmick,[b] David Xi Cao,[a] Geza Szigethy,[a] Joseph W. Ziller,[a] Matthew P. Shores[b] and Alan F. Heyduk,*[a]*

[a]Department of Chemistry, University of California, Irvine, California, 92697, USA. E-mail: aheyduk@uci.edu; Tel: +1-949-824-8806.

[b]Department of Chemistry, Colorado State University, Fort Collins, Colorado, 80523, USA. E-mail: matthew.shores@colostate.edu; Tel: +1-970-491-7235

Table of Contents **Page**

Experimental Procedures S2

Table S1. X-ray diffraction data-collection and refinement parameters for complexes $Fe_2(ONO)_3$, $FeZn(ONO)_3$, and $(ONO^{sq\bullet})Zn(py)_2$. S5

EPR and UV-vis-NIR spectra of $Fe_2(ONO)_3$, $FeZn(ONO)_3$, and $(ONO^{sq\bullet})Zn(py)_2$ S6

Discussion of alternative spin models for $FeZn(ONO)_3$ S9

Discussion of alternative spin models for $Fe_2(ONO)_3$ S13

References S15

Experimental Procedures

General Considerations. All compounds and reactions reported below show various levels of air- and moisture-sensitivity, so all manipulations were carried out using standard vacuum-line, Schlenk-line and glovebox techniques. Solvents were sparged with argon before being deoxygenated and dried by passage through Q5 and activated alumina columns, respectively. To test for effective oxygen and water removal, aliquots of each solvent were treated with a few drops of a purple solution of sodium benzophenone ketyl radical in THF. Potassium hydride (Alfa Aesar) was washed with dry degassed pentane to remove the oil prior to usage. The ligand precursors $(ONO^{cat})H_3$ and $K(ONO^{q})$ were prepared according to published procedures.[1,2] The homoleptic metal complexes $M(ONO)_2$ (M = Fe, Zn) were prepared according to the method of Balch.[3] The iron complexes $(ONO^{q})FeCl_2$ and $(ONO^{cat})Fe(py)_3$ were prepared according to published procedures.[4]

Physical Measurements. Electronic absorption spectra were recorded on THF solutions of the complex at ambient temperature (20-24 °C) using a PerkinElmer Lamda 900 UV-vis-NIR spectrometer and one-centimeter path-length cells. Perpendicular-mode X-band EPR spectra were collected using a Bruker EMX spectrometer equipped with an ER041XG microwave bridge an ER4116DM dual-mode cavity, and an Oxford Instrument liquid He quartz cryostat. EPR spectra were recorded using a microwave frequency of 9.64 GHz and power of 2.0 mW. Electrospray ionization mass spectrometry (ESI-MS) was performed at the Mass Spectrometry Facility at University of California, Irvine. Elemental analyses were performed on a PerkinElmer series II 2400 CHNS analyzer.

Crystallographic Methods. X-ray diffraction data for all complexes were collected on single crystals mounted on a glass fiber and coated with oil. Data were acquired using a Bruker SMART APEX II diffractometer and Mo Kα radiation (λ = 0.71073 Å). The APEX2[5] program package was used to determine unit-cell parameters and for data collection. The raw frame data were processed using SAINT[6] and SADABS[7] to yield the reflection data file. Subsequent refinement cycles were carried out using the SHELXTL program suite.[8] Analytical scattering factors for neutral atoms were used throughout the analyses.[9] Hydrogen atoms were added using a standard riding model.

- For $Fe_2(ONO)_3$: The diffraction symmetry was $2/m$ and the systematic absences were consistent with the monoclinic space group $P2_1/c$ that was later determined to be correct. The acetonitrile molecule occupies a solvent void and was refined isotropically.
- For $FeZn(ONO)_3$: The diffraction symmetry was $2/m$ and the systematic absences were consistent with the monoclinic space group $P2_1/c$ that was later determined to be correct. Methyl groups C(26)-C(28), C(40)-C(42) and C(82)-C(84) were disordered and included using multiple components with partial site-occupancy-factors.
- For $(ONO^{sq\bullet})Zn(py)_2$: The diffraction symmetry was $2/m$ and the systematic absences were consistent with the monoclinic space groups $P2_1$ and $P2_1/m$. It was later determined that space group $P2_1$ was correct. There were two molecules of the formula-unit, one molecule of pyridine solvent and one molecule of acetonitrile solvent present (Z = 4). The absolute structure was assigned by refinement of the Flack parameter.[10]

ORTEP diagrams were generated using ORTEP-3 for Windows.[11] Diffraction data for $Fe_2(ONO)_3$, $FeZn(ONO)_3$, and $(ONO^{sq\bullet})Zn(py)_2$ are given in **Table S1**.

Magnetic methods. Magnetic susceptibility measurements were collected using a Quantum Design MPMS XL SQUID magnetometer. Powdered microcrystalline samples were loaded into polyethylene bags, sealed in the glovebox, inserted into a straw, and transported to the SQUID magnetometer under a nitrogen atmosphere. The presence of ferromagnetic impurities was checked by a variable field analysis (0 to 10 kOe) of the magnetization data at 100 K, where linearity of the M vs H plot indicates the absence of any significant impurities. DC magnetic susceptibility data were collected at temperatures ranging from 2 to 300 K at applied fields of 1 kOe. Multiple full-range susceptibility runs from the same sample batch were used to ensure reproducibility with the largest $\Delta\chi_M T$ of *ca.* 0.14 cm^3 K mol^{-1} was measured for $Fe_2(ONO)_3$ at 300 K (where $\chi_M T$ is 7 cm^3 K mol^{-1}). Magnetization measurements were collected at temperatures ranging from 2 to 35 K and at applied fields of 10, 20, 30, 40, and 50 kOe. Data were corrected for the diamagnetic contributions of the sample holder and bag by subtracting empty containers. Diamagnetic corrections for the sample were calculated from Pascal's constants.[12] Theoretical fits to the susceptibility data for $FeZn(ONO)_3$ and $Fe_2(ONO)_3$ were obtained using a relative error minimization routine in julX.[13] For $Fe_2(ONO)_3$, the spin Hamiltonian took the form,

$$\widehat{H} = -2J\hat{S}_1 \bullet \hat{S}_2$$

An alternate spin Hamiltonian for the anisotropy tensors bears the form,

$$\widehat{H} = \sum_{i=1}^{n} g\beta\overline{S_i} \bullet \overline{H} + \sum_{i=1}^{n}\left[D_i S_{z,i}^2 - \frac{1}{3D_i S_i\left(S_i+1\right)} + E_i\left(S_{x,i}^2 - S_{y,i}^2\right)\right]$$

An additional fit was performed on $Fe_2(ONO)_3$ using Anisofit[14] and a spin Hamiltonian of the form,

$$\widehat{H} = D_i\hat{S}_z^2 + E\left(\hat{S}_z^2 + \hat{S}_y^2\right) + g_{iso}\mu_B S \bullet H$$

Additional fits were attempted using the program PHI[15] to determine the interactions between the spin centers.

Synthesis of $(ONO^{sq\bullet})Zn(py)_2$. A 10 mL pyridine solution containing 125 mg of $Zn(OAc)_2$ (0.681 mmol, 1 equiv.) was treated with $(ONO^{sq\bullet})K_2$ (0.680 mmol, 1 equiv.; generated from a 1:1 mixture of $(ONO^{cat})K_3$ and $(ONO^{q})K$) dissolved in pyridine and was stirred at ambient glovebox temperature for 18 hours. The reaction mixture was filtered and the solid byproduct was washed with an additional 15 mL of pyridine. The washings were added to the mother liquor and the dark green solution was concentrated under reduced pressure. The solution was layered with acetonitrile and stored at –35 °C resulting in the precipitation of dark green crystals. Yield: 312 mg (71%).

Anal. Calc. (Found) for $C_{38}H_{50}N_3O_2Zn$ (%): C: 70.63 (70.14); 7.80 (7.87); N: 6.50 (6.23). UV-vis-NIR (THF) λ_{max} / nm (ε / M^{-1} cm^{-1}): 398 (27,900), 956 (10,400). MS (ESI+) *m/z*: 646.3 (M+).

Synthesis of $Fe_2(ONO)_3$. *Method A*: A mixture of 30.5 mg potassium metal (0.780 mmol, 2 equiv.) and 77.5 mg graphite (6.45 mmol, 16 equiv.) was heated under vacuum in the presence of a Teflon stir bar with constant agitation until the solids were finely divided and bronze in color.

This KC_8 was suspended in 5 mL of THF and frozen in a liquid-nitrogen cold well. To this mixture was added a barely-thawed, dark-green solution containing 352 mg $Fe(ONO)_2$ (0.390 mmol, 1 equiv.) dissolved in 10 mL of THF. The resultant brown-black mixture was stirred at room temperature for 1 hour. The mixture was refrozen and upon thawing, 214 mg of $(ONO^{q})FeCl_2$ (0.390 mmol, 1 equiv.) dissolved in 10 mL of THF was added. The mixture was stirred overnight at ambient temperature, during which time it turned dark purple. The reaction mixture was filtered to remove graphite and the solvent was removed from the mother liquor under reduced pressure to yield a glassy, black foam. Addition of 3 mL of acetonitrile to this residue and cooling to –35 °C yielded the product as a dark purple powder (243 mg over two crops, 77% yield).

Method B: A 10 mL toluene solution containing 131 mg of $Fe(ONO)_2$ (0.146 mmol, 1 equiv.) and 105 mg of $(ONO^{cat})Fe(py)_3$ (0.146 mmol, 1 equiv.) was stirred at ambient temperature for 20 hours. The volatiles were removed under reduced pressure and the solid residue was taken up in a minimal amount of toluene, filtered, layered with acetonitrile and stored at –35 °C. The product was precipitated as a dark purple powder. Yield: 177 mg (88%).

Anal. Calc. (Found) for $C_{84}H_{121}N_3O_6Fe_2$ (%): C, 73.08 (72.93); H, 8.83 (8.74); N, 3.05 (2.95).UV-vis-NIR (THF) λ_{max} / nm (ε / M^{-1} cm^{-1}): 318 (44,900), 350 (52,400), 506 (17,600), 890 (23,700). MS (ESI+) *m/z*: 1378.75 (M+).

Synthesis of FeZn[ONO]$_3$. A 10 mL toluene solution containing 132 mg of $Fe(ONO)_2$ (0.146 mmol, 1 equiv.) and 94.5 mg of $(ONO^{sq\bullet})Zn(py)_2$ (0.146 mmol, 1 equiv.) was stirred at ambient glovebox temperature for 20 hours. Upon removal of volatiles under reduced pressure, the resulting dark green residue was redissolved in toluene. Diffusion of acetonitrile into the toluene solution at –35 °C resulted in the formation of a dark green precipitate. Yield: 150.6 mg (74%).

Anal. Calc. (Found) for $C_{84}H_{120}FeN_3O_6Zn$ (%): C, 72.63 (72.35); H, 8.71 (9.15); N, 3.02 (2.93). UV-vis-NIR (THF) λ_{max} / nm (ε / M^{-1} cm^{-1}): 354 (20,200), 438 (14,000), 746 sh (21,400), 806 (24,400). MS (ESI+) *m/z*: 1386.75 (M+).

Table S1. X-ray diffraction data-collection and refinement parameters for complexes $Fe_2(ONO)_3$, $FeZn(ONO)_3$, and $(ONO^{sq\bullet})Zn(py)_2$.

	$Fe_2(ONO)_3$ • ½ CH_3CN	$FeZn(ONO)_3$	$(ONO^{sq\bullet})Zn(py)_2$ • ½ C_5H_5N • ½ CH_3CN
empirical formula	$C_{85}H_{121.5}N_{3.5}O_6Fe_2$	$C_{84}H_{120}N_3O_6FeZn$	$C_{41.5}H_{54}N_4O_2Zn$
formula weight	1400.06	1389.04	706.25
crystal system	monoclinic	monoclinic	monoclinic
space group	$P2_1/c$	$P2_1/c$	$P2_1$
T / K	88(2)	143(2)	88(2)
a / Å	15.5180(10)	15.5857(8)	14.208(3)
b / Å	20.0893(13)	20.1236(11)	20.222(4)
c / Å	26.9179(18)	26.8587(14)	14.512(3)
α / deg	90	90	90
β / deg	104.1755(9)	104.2326(7)	108.954(3)
γ / deg	90	90	90
V / Å^3	8136.0(9)	8165.4(7)	3943.6(12)
Z	4	4	4
Refl. collected	87960	87548	46059
data/restraints/parameters	16658/0/916	16711/0/985	17365/1/899
R1 ($I > 2\sigma_I$)^a	0.0412	0.0429	0.0435
wR2 (all data)^b	0.1059	0.1300	0.1005
GOF^c	1.013	1.035	1.074

^{a}R1 = $\Sigma||F_o| - |F_c||/\Sigma|F_o|$; ^{b}$w$R2 = $[\Sigma[w(F_o^2 - F_c^2)^2]/\Sigma[w(F_o^2)^2]]^{1/2}$; ^{c}GOF = $[\Sigma w(|F_o| - |F_c|)^2/(n - m)]^{1/2}$

EPR and UV-vis-NIR spectra of $Fe_2(ONO)_3$, $FeZn(ONO)_3$, and $(ONO^{sq\bullet})Zn(py)_2$

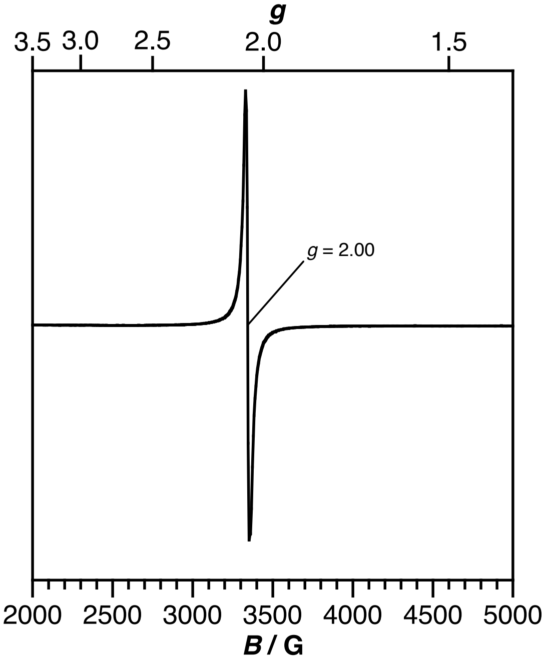

Figure S1. X-Band EPR spectrum of $(ONO^{sq\bullet})Zn(py)_2$ dissolved in pyridine at 77K.

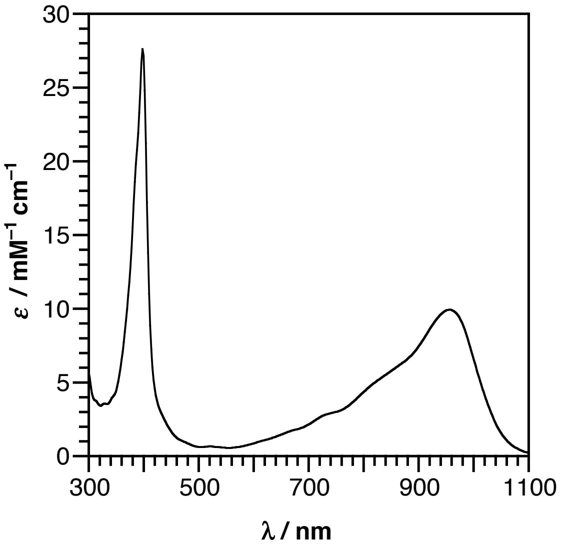

Figure S2. UV-vis-NIR absorption spectrum of $(ONO^{sq\bullet})Zn(py)_2$ dissolved in THF.

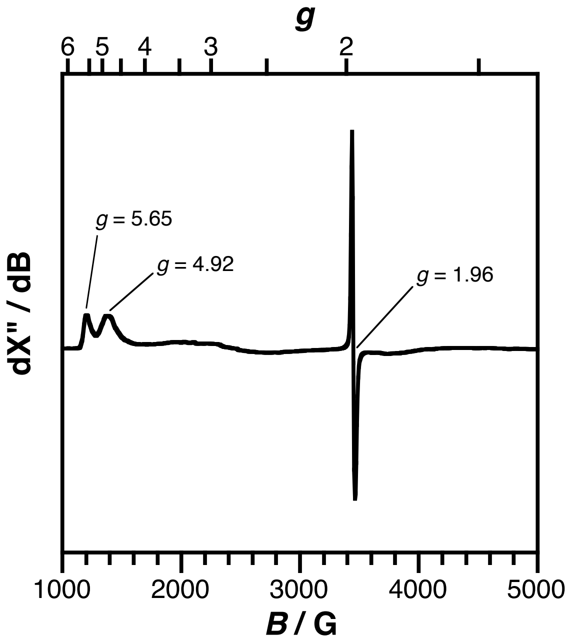

Figure S3. X-Band EPR spectrum of FeZn(ONO)$_3$ dissolved in THF at 4 K.

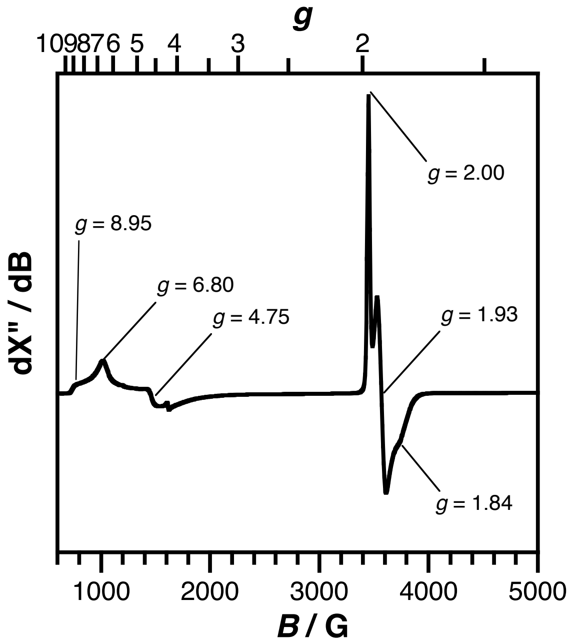

Figure S4. X-Band EPR spectrum of Fe$_2$(ONO)$_3$ dissolved in THF at 4 K.

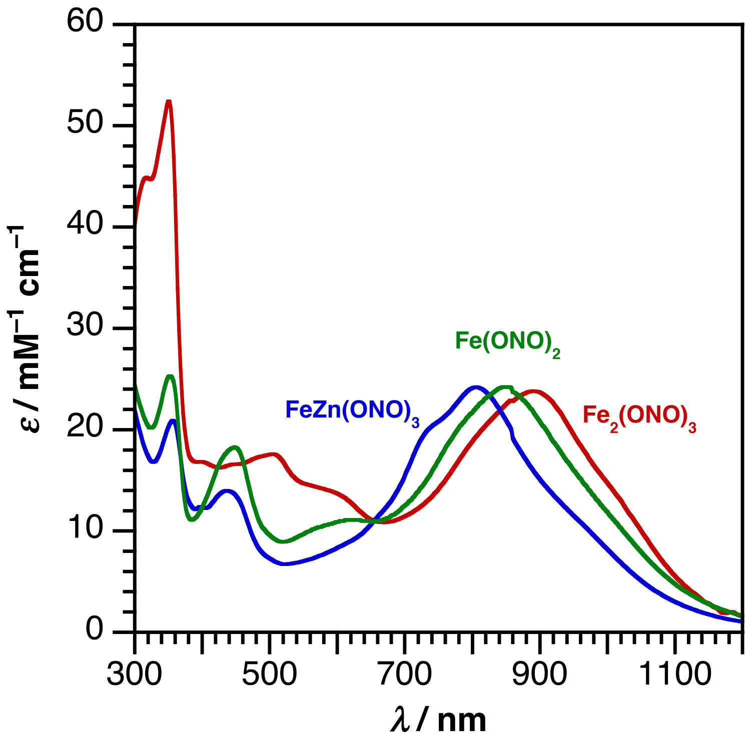

Figure S5. UV-vis-NIR absorption spectrum of $Fe_2(ONO)_3$, $FeZn(ONO)_3$, and $Fe(ONO)_2$ dissolved in THF.

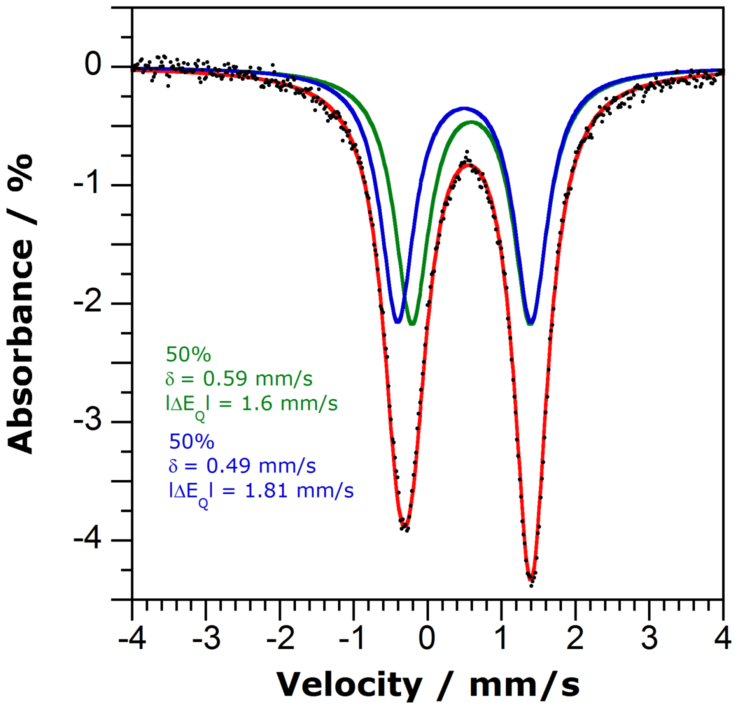

Figure S6. Mössbauer spectrum of $Fe_2(ONO)_3$ collected at 80 K.

Discussion of alternative spin models for FeZn(ONO)$_3$

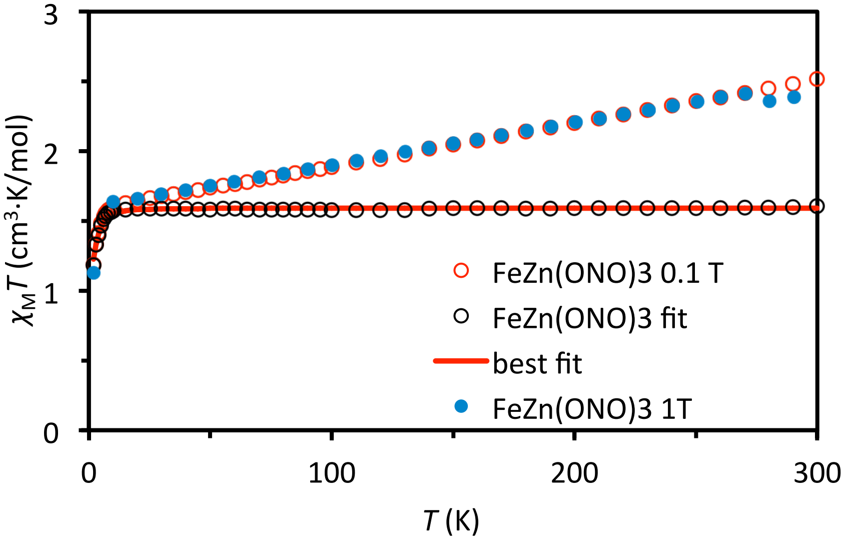

Figure S7. Solid state temperature dependence of magnetic susceptibility for FeZn(ONO)$_3$ collected at an applied fields of 1000 and 10,000 Oe: raw data and data subtracted for TIP are shown as red and black circles, respectively; the red line represents the best fit (model *b* in **Table S2**) to the data from julX.

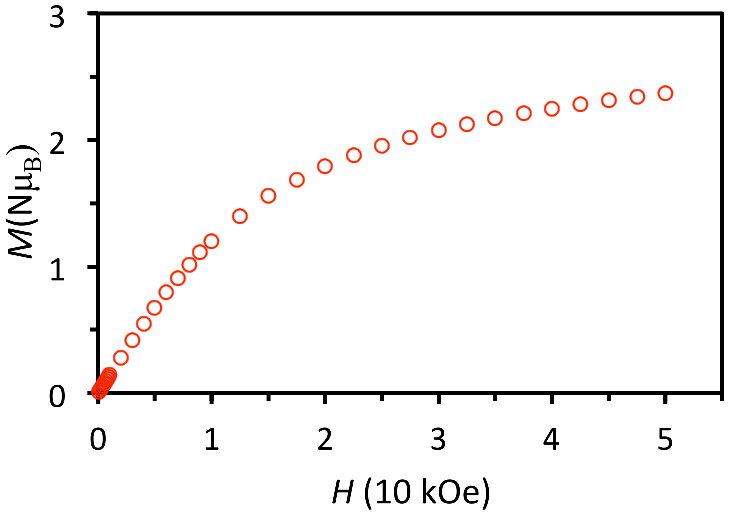

Figure S8. *M* vs. *H* for FeZn(ONO)$_3$ ranging from 0 to 50 kOe at 1.8 K. The largest magnetization value is 2.37 μ_B at 50 kOe. An applied dc field of 50 kOe is not large enough to fully populate the ground state of FeZn(ONO)$_3$.

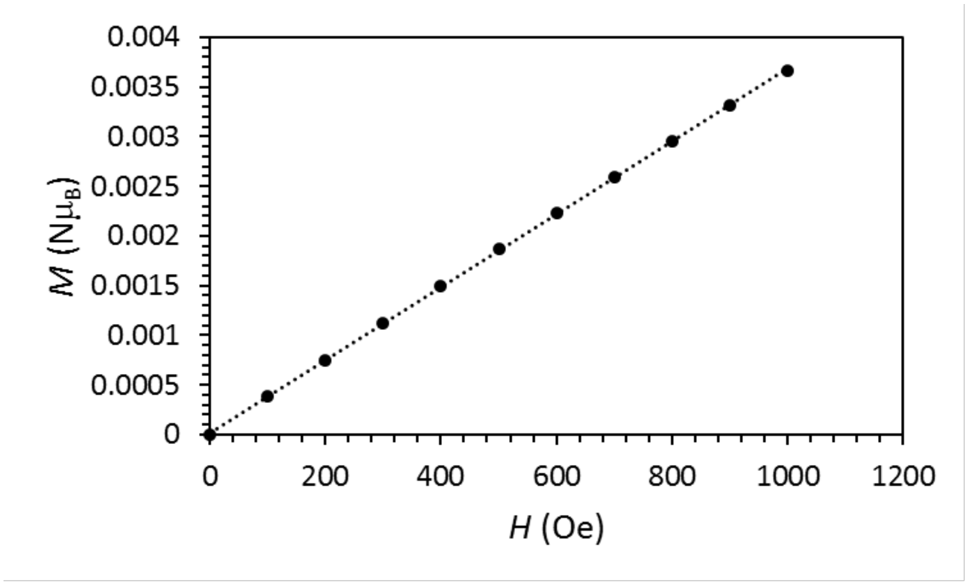

Figure S9. *M* vs. *H* for $FeZn(ONO)_3$ ranging from 0 to 1 kOe at 100 K where $R^2 = 0.9999$.

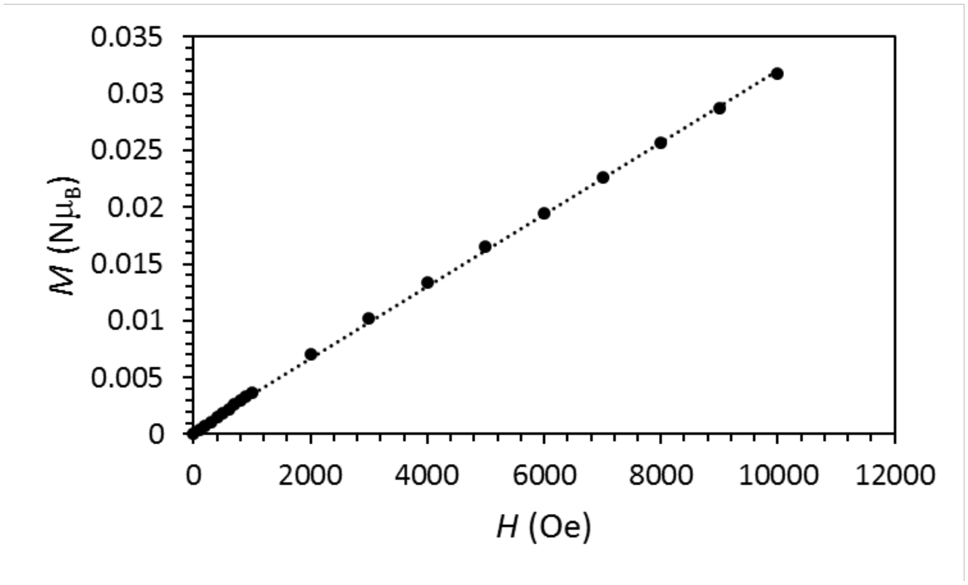

Figure S10. *M* vs. *H* for $FeZn(ONO)_3$ ranging from 0 to 10 kOe at 100 K where $R^2 = 1$.

Table S2. Alternative spin model parameters for fitting the magnetic susceptibility of $FeZn(ONO)_3$ to an $S = 3/2$ model using julX.[13]

entry	g	TIP / 10^{-6} cm^3 mol^{-1}	D / cm^{-1}	E / cm^{-1}	Θ / cm^{-1}	f_{sum}
a	1.86	2810	n/a	n/a	–0.60	0.0213
b (best)	1.84	2910	–2.96	n/a	–0.058	0.0114
c	1.84	2910	+2.46	–0.39	–0.058	0.0114
d	1.84	2930	–3.02	0.19	n/a	0.0116

A model that excludes anisotropy parameters but incorporates intermolecular interactions (entry a in **Table S2**) gives an inferior fit to the data, both visually – especially at low temperatures – and by the relatively large f_{sum} value. Including an axial anisotropy (D) term but neglecting a rhombic anisotropy (E) term (entry b) gives a better f_{sum} value, and is used in Figure 3. A similar f_{sum} value is obtained by adding the rhombic anisotropy E parameter (entry c) but it was not chosen because of the ambiguity related to the relative signs of anisotropy parameters D and E. Therefore the model chosen as the best (entry b) includes both axial anisotropy parameter (D) and intermolecular coupling (Θ). Removing Θ (entry d) gives a similar quality fit as b and c, and slightly changes the absolute values of the anisotropy parameters, but with a slightly larger f_{sum} value; note that the change of sign for D is not considered a significant problem since the signs of the anisotropy parameters cannot be reliably determined from bulk magnetic measurements.

Two models were used to estimate antiferromagnetic coupling (J) in $FeZn(ONO)_3$. The first model contained an $S = 2$ ferrous center and a single $S = ½$ $(ONO^{sq\bullet})^-$ ligand. Inputting fixed values for TIP (3000×10^{-6} cm^3 mol^{-1}) and Θ (–0.09 cm^{-1}) into the program PHI,[15] afforded g = 1.88 and J = –260.19 cm^{-1} with a residual (equivalent to f_{sum}) of 0.022 (**Figure S11**, left). An alternative model, which included an $S = 5/2$ ferric center and two $S = ½$ $(ONO^{sq\bullet})^-$ ligands, and used fixed values for TIP (2900×10^{-6} cm^3 mol^{-1}) and Θ (–0.09 cm^{-1}), afforded g = 1.89 and three different antiferromagnetic coupling values ($J_{(Fe\text{-}ligand1)}$ = –164.89 cm^{-1}, $J_{(Fe\text{-}ligand2)}$ = –0.99 cm^{-1}, $J_{(ligand1\text{-}ligand2)}$ = –3.28 cm^{-1}) with a residual of 0.019 (**Figure S11**, right). Even though the residual improved slightly in the second model, the low value of Fe-ligand2 antiferromagnetic coupling parameter compared to Fe-ligand1 is not reasonable given the nominally identical SOMO overlap between the iron center and the two $(ONO^{sq\bullet})^-$ ligands. Combined with the spectroscopic data (UV-vis-NIR) of this compound (**Figure S5**), the ferrous model with one radical center is more convincing than the ferric model.

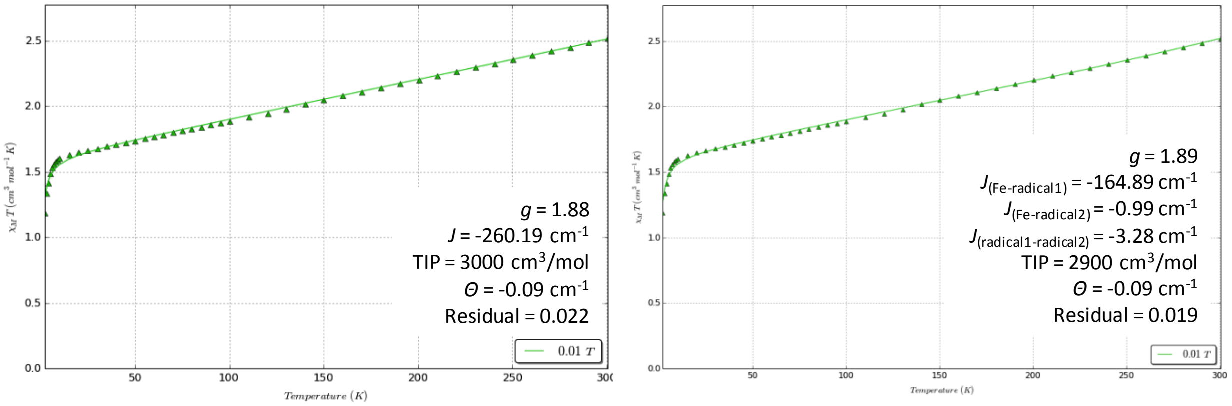

Figure S11. Fits of temperature-dependent dc susceptibility data of $FeZn(ONO)_3$ complex to two models: (*left*) an $S = 2$ iron(II) coupled to a single $S = ½$ radical ligand; (*right*) an $S = ^5/_2$ iron(III) and two $S = ½$ radical ligands. The TIP and Θ values were fixed to obtain the best-fit parameters.

Discussion of alternative spin models for $Fe_2(ONO)_3$

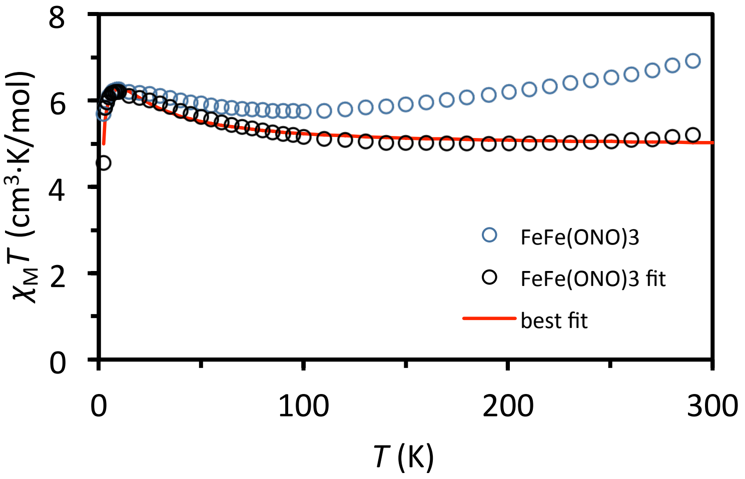

Figure S12. Temperature-dependent dc susceptibility data for $Fe_2(ONO)_3$ (blue circles) collected at an applied field of 1000 Oe, and the same data subtracted for TIP (black circles) with the best fit shown as the red line. *Note: the best fit assumes that each iron center interacts very strongly with one radical, and then the resulting* S = 2 *and* S = $^3/_2$ *fragments interact with each other in a weaker fashion. The true model should include interaction of the bridging radical with both iron centers, thus some of the curvature of the data is not captured in the simple model.*

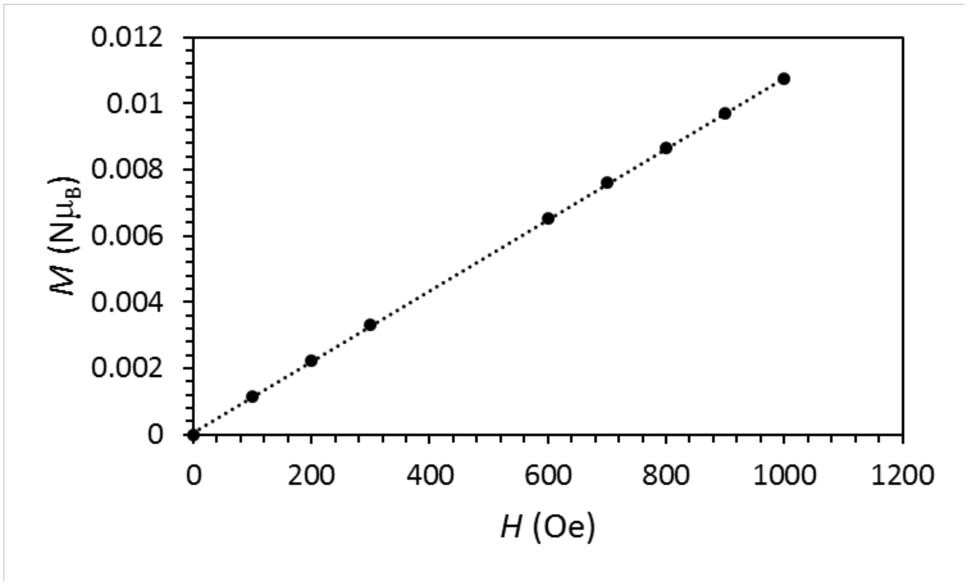

Figure S13. *M* vs. *H* for $FeFe(ONO)_3$ ranging from 0 to 1 kOe at 100 K where $R^2 = 0.9999$. The 400 and 500 Oe data points were omitted due to a centering difficulty on the instrument.

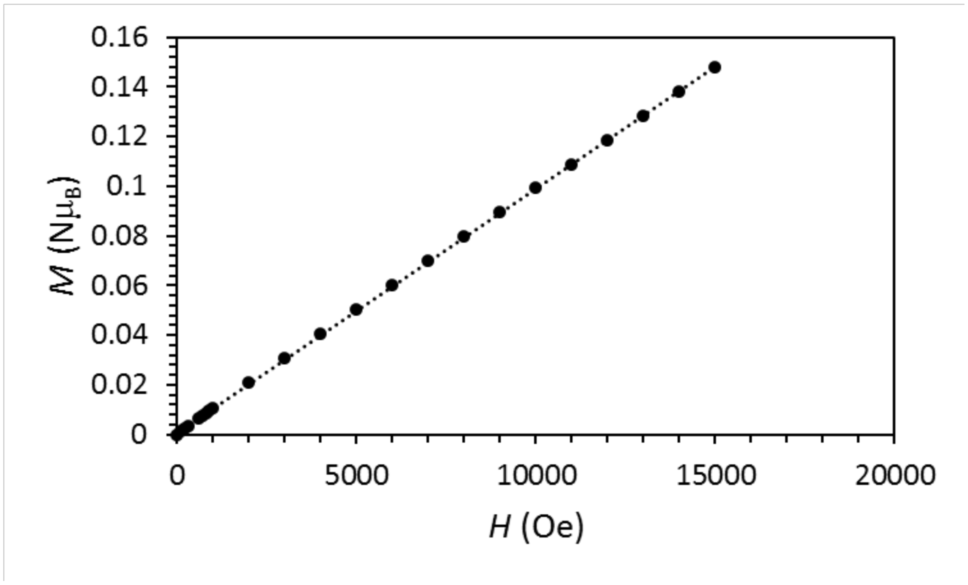

Figure S14. *M* vs. *H* for $FeFe(ONO)_3$ ranging from 0 to 15 kOe at 100 K where $R^2 = 0.9999$. The 400 and 500 Oe data points were omitted due to a centering difficulty on the instrument.

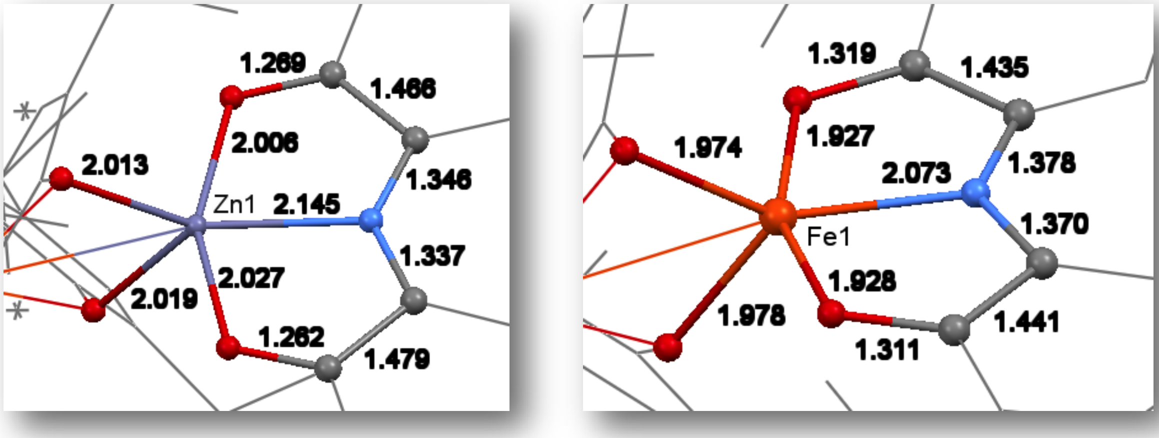

Figure S15. Comparison of M interactions with the non-bridging ONO ligand for $FeZn(ONO)_3$ (*left*) and $Fe_2(ONO)_3$ (right). The presence of radical on the semiquinonate ligand increases the electron density on the *non*-bridging ligand, thus increasing the C–N and C–O bond distances.

References

1. P. Chaudhuri, M. Hess, T. Weyhermüller and K. Wieghardt, *Angew. Chemie Int. Ed.,* 1999, **38**, 1095–1098
2. G. Szigethy, D. W. Shaffer and A. F. Heyduk, *Inorg. Chem.,* 2012, **51**, 12606-12618.
3. A. Y. Girgis and A. L. Balch, *Inorg. Chem.,* 1975, **14**, 2724–2727.
4. J. L. Wong, R. H. Sánchez, J. G. Logan, R. A. Zarkesh, J. W. Ziller and A. F. Heyduk, *Chem. Sci.,* 2013, **4**, 1906-1910.
5. APEX2 Version 2013.6-2, Bruker AXS, Inc.; Madison, WI 2013.
6. SAINT Version 8.32b, Bruker AXS, Inc.; Madison, WI 2012.
7. Sheldrick, G. M. SADABS, Version 2012/1, Bruker AXS, Inc.; Madison, WI 2012.
8. Sheldrick, G. M. SHELXTL, Version 2014/7, Bruker AXS, Inc.; Madison, WI 2014.
9. *International Tables for Crystallography*, A. J. C. Wilson, Ed.; Kluwer Academic, Dordrecht, Netherlands, 1992, Vol. C.
10. H. D. Flack, S. Parsons and T. Wagner, *Acta. Cryst.,* 2013, B69.
11. L. J. Farrugia, *J. Appl. Crystallogr.*, 1997, **30**, 565.
12. G. A. Bain and J. F. Berry, *J. Chem. Educ*., 2008, **85**, 532
13. E. Bill, julX, Version 1.41, Max Planck Institute for Bioinorganic Chemistry; Mulheim an der Ruhr, Germany, 2008.
14. M. P. Shores, J. J. Sokol and J. R. Long, *J. Am. Chem. Soc*., 2002, **124**, 2279.
15. N. F. Chilton, R. P. Anderson, L. D. Turner, A. Soncini and K. S. Murray, *J. Comput. Chem*., 2013, **34**, 1164
